# Enhancing ECG disease detection accuracy through deep learning models and P-QRS-T waveform features

**DOI:** 10.1371/journal.pone.0325358

**Published:** 2025-06-10

**Authors:** Rida Nayyab, Asim Waris, Iqra Zaheer, Muhammad Jawad Khan, Fawwaz Hazzazi, Muhammad Adeel Ijaz, Hassan Ashraf, Syed Omer Gilani

**Affiliations:** 1 Department of Biomedical Engineering and Sciences, School of Mechanical and Manufacturing Engineering, National University of Sciences and Technology (NUST), Islamabad, Pakistan; 2 Department of Electrical Engineering, School of Engineering, Prince Sattam Bin Abdul Aziz University, Saudi Arabia; 3 Department of Electrical Engineering, College of Engineering, Prince Sattam Bin Abdul Aziz University, Al-Kharj, Saudi Arabia; 4 Laboratory of Movement Analysis (LAM-Motion Lab), University of Liege, Liege, Belgium; 5 Department of Electrical, Computer, and Biomedical Engineering, Abu Dhabi University, Abu Dhabi, United Arab Emirates; VIT-AP Campus, INDIA

## Abstract

Cardiovascular diseases (CVDs) have surpassed cancer and become the major cause of death worldwide. An electrocardiogram (ECG) is a non-invasive and quicker method for diagnosing abnormal heart conditions. While research has extensively focused on ECG analysis for disease classification, it has been primarily directed toward binary classification or classification of Arrhythmias, highlighting the dire need for detailed classification models. This study utilises the extensive PTB-XL database ECG records to develop a robust method for classifying various heart abnormalities. The data with unique labels is filtered through the Butterworth bandpass filter and Discrete Wavelet Transform (DWT) db-8. The R-peaks of the clean signal were used to detect the subsequent morphological features, i.e., P-QRS-T intervals and amplitudes. The feature set was balanced using the Synthetic Minority Oversampling Technique for Nominal and Continuous (SMOTE-NC) and fed into Convolutional Neural Network (CNN) and Deep Neural Network (DNN) with 5-fold cross-validation. The models classified the ECG records into one normal and four abnormal classes: Conduction Disturbance (CD), Myocardial Infarction (MI), Hypertrophy (HYP), and ST-T Changes (STTC). Performance metrics such as F1 score, recall, precision, and accuracy were evaluated for each model. The CNN model achieved a mean accuracy of 81% ± 0.03, while the DNN model achieved a mean accuracy of 84% ± 0.01. One key finding is that Hypertrophy (HYP) was consistently classified with up to 98% accuracy. Thus, the study demonstrates the effectiveness of combining advanced signal processing and deep learning techniques for precise multi-class heart disease classification using P-QRS-T features, paving the way for future real-time clinical applications.

## Introduction

Cardiovascular diseases (CVDs) are a broad category of diseases affecting the heart and blood vessels. In 2021, the WHO estimated that CVD took the lives of 20.5 million people, and they predict this number will double by 2030 [1]. CVDs can cause blockage in the heart vessels or blood clots that result in cerebral or cardiac ischemic necrosis, causing heart attacks and strokes. It accounts for one-half of the CVD cases [2].

Early detection of these heart conditions allows timely intervention, decreasing human mortality rates. Since most heart conditions are coupled with cardiac arrhythmias, known as abnormal heartbeats, they are easily reflected on the electrocardiograph (ECG) [3]. ECG is a noninvasive and quicker approach to reliably detecting CVDs [4]. It represents the changing electrical potential of the heart during a cardiac cycle. In a time series, each deflection represents the electric activity of the heart and muscle contraction [5].

Morphological features are the critical cardiac features of the ECG signal. They include the amplitudes and time intervals of the P-QRS-T waveform for each beat. Cases like ischemia are reflected on ECG with ST-segment deviation, while conduction disturbance due to blockage or hypertrophy is indicated by prolonged PR intervals or abnormal QRS [6].

However, the ECG signal is accompanied by high noise. Electrodes are placed on different body tissues to measure their potential differences. Thus, a slight movement can introduce a baseline drift, a low-frequency interference. Similarly, muscle movement or breathing can introduce noise in the signal that is difficult to extract from the actual signal. ECG is also highly complex and consists of individual variability. Thus, signal interpretation becomes tiresome, introducing subjective uncertainty and human error [7].

Computer-aided analysis yields faster, more accurate, and more reliable results. In recent years, Machine Learning (ML) models, particularly algorithms known as Deep Learning (DL) models, have revolutionized the way ECG patterns are analysed. It recognizes subtle patterns and variations in ECG signals that might be missed by manual analysis and enhances diagnostic accuracy and reliability. Different DL models like ResNet, Inception V3, Gated Recurrent Unit (GRU), and Long Short-Term Memory (LSTM) are used for ECG classification that has high accuracy [8]. However, most existing studies have focused on binary classification, distinguishing between normal and abnormal heart conditions, limiting their utility in diagnosing more complex cardiovascular diseases [9]. This research addresses this limitation by focusing on the multi-class classification of ECG data, a relatively underexplored area.

In recent times, PTB-XL, one of the largest open databases for ECG signals, allowed researchers to work on five significant super-classes, i.e., Normal (NORM), Myocardial Infraction (MI), Hypertrophy (HYP), Conduction Disturbance (CD), ST-T changes (STTC) and their 20 sub-classes. Smigiel et al.. worked on 2, 5, and 20 classes of CVDs from the PTB-XL dataset. They used the entropy-based features to train the CNN model; however, its overall weight was increased by two to seven folds with 76% accuracy, reducing its scalability [3]. Palczynski et al.. used R-peaks of the QRS complex to train FSL network and Softmax-based counterparts and achieved an accuracy range of 75.1% to 80.2% [10]. Tadejko [11] used mathematical morphology with Self-organizing Maps (SOM) and Learning Vector Quantization (LVQ), focusing on RR intervals for ECG feature extraction using the MIT-BIH dataset to classify arrhythmias only. Chaos theory was implemented by Alan and Nikola [12] for ECG classification, which resulted in no significant detection. Chouhan and Mehta [13] focused on QRS complex detection for RR interval extraction on binary class. Similarly, Olvera [14] focused solely on the ST segment for classification. Additionally, studies like Noman and Hammad [15] employed machine learning techniques for binary classification using a limited set of P-QRS-T features but did not fully leverage deep learning for multi-class classification.

Building on the foundation established by previous studies, this research addresses several critical gaps in ECG-based disease classification. While studies such as those by Smigel et al. [3] and Palczynski et al. [10] have advanced understanding of CVD classification, their focus on binary classification or small subsets of the dataset limited their utility in tackling the broader and more complex spectrum of cardiovascular diseases (CVDs). Moreover, techniques such as entropy-based features and R-peak detection have shown promise but fail to capture the full range of critical features necessary for distinguishing between multiple classes of CVDs.

This study overcomes these limitations by preprocessing the ECG signals using both Butterworth bandpass filtering and Discrete Wavelet Transform (DWT). These two methods were combined to remove both high-frequency noise and minute-level distortions, ensuring that the ECG signals are clear and reliable for classification. This is especially important when working with a wide range of diseases, where the accuracy of detecting subtle variations in the P-QRS-T segments can make a significant difference in diagnosis. Thus, the dual approach ensures that all critical features of the ECG signal are preserved, enabling better classification across five broad disease classes.

Furthermore, while advanced deep learning models like ResNet and Inception V3 have been used in ECG classification with good results, this study demonstrates that even simpler models like CNNs and DNNs can achieve high accuracy in this complex task. The decision to use these simpler models was driven by the need to balance computational efficiency with classification accuracy. In contrast to more advanced models like ResNet or Inception V3, which require significant computational resources due to their deep and complex architectures, CNNs and DNNs offer a more resource-efficient alternative for multi-class ECG classification. While CNNs and DNNs are still computationally intensive, their relatively simpler structure allows for more efficient training and deployment without the high costs associated with deeper, more complex networks. These models can effectively handle the classification of multiple cardiovascular conditions while maintaining accuracy and scalability, making them an attractive choice for real-time clinical applications.

Class imbalance in PTB-XL dataset skewed model performance towards more prevalent classes. By implementing SMOTE-NC for class balancing, this study not only mitigates this issue but also enhances the model’s ability to detect and classify underrepresented conditions such as Hypertrophy (HYP) and Conduction Disturbance (CD), which previous studies like Palczynski et al. [10] have shown to be difficult to classify accurately.

Employing a robust 80−20% train-test split and 5-fold cross-validation, the models are evaluated on various performance metrics, including accuracy, precision, recall, and F1 score. This comprehensive approach offers new insights in significant advancement of ECG-based disease detection, offering a robust, multi-class classification framework that is computationally efficient yet highly accurate. By integrating advanced preprocessing techniques, class balancing, and deep learning models, it paves the way for real-time clinical applications that can improve diagnostic workflows and support early intervention in cardiovascular diseases.

## Materials and methods

**Fig 1:**
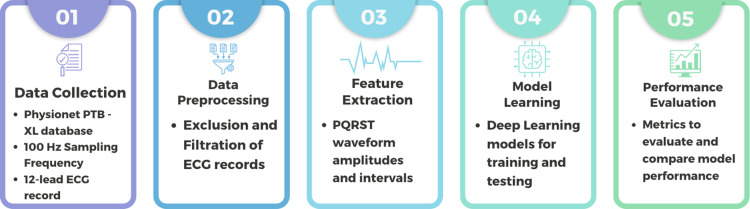
Machine learning pipeline for multi-class classification.

The diagrammatic representation of the study is given in Fig 1.

### A. Dataset

**Fig 2:**
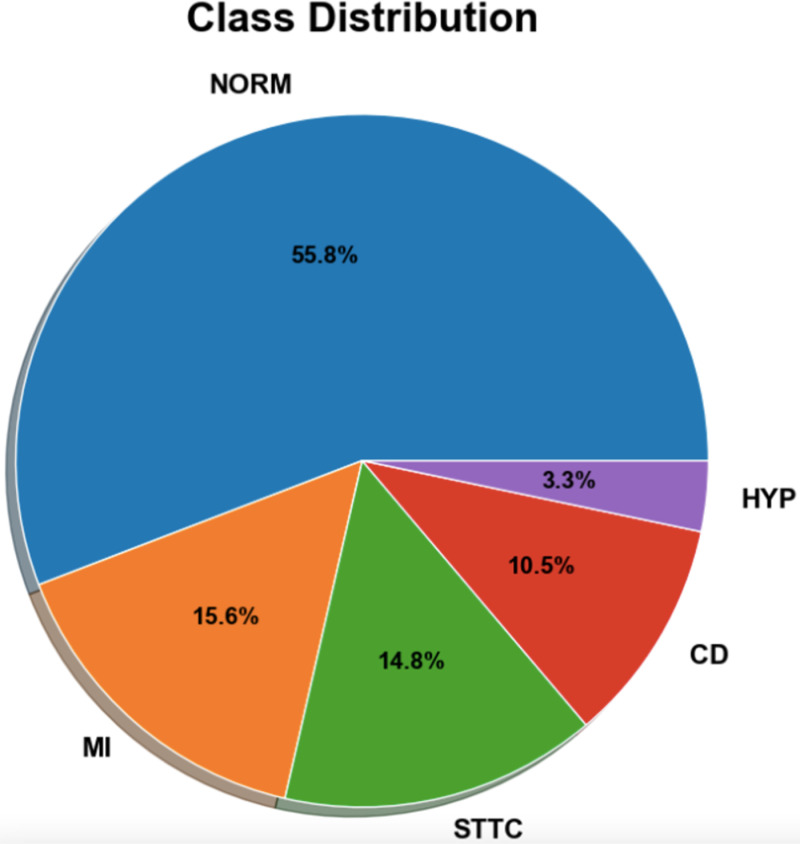
Distribution of ECG samples in the dataset.

The research utilizes the ECG signals from the open database PTB-XL (16). This database consists of 21,837 clinical signals recorded from a traditional 12-lead ECG. PTB-XL provides data sampled on 500 and 100 Hz sampling frequencies, and for this study, a 100 Hz sampling frequency was chosen. The data is derived from 18,885 patients with a balanced ratio of gender, i.e., 52% males and 48% females. The dataset has five major super-classes: (i) Normal (NORM), (ii) Myocardial Infraction (MI), (iii) Hypertrophy (HYP), (iv) Conduction Disturbance (CD), and (v) ST-T changes (STTC) with approximately 20 subclasses. (Fig 2).

### B. Data filtering

All the ECG records in the dataset are not labeled, and some are labeled without 100% certainty. These records were filtered out. This process was critical to ensure data consistency and integrity. The records with multiple labels were ruled out in the next step, leaving a final dataset of 17,232 ECG records. This ensured that only unambiguous data points were included in the final dataset, avoiding potential bias or confusion in model training and evaluation. The database with 100 Hz sampling frequency was used for the analysis.

### C. Data Pre-processing

The data is corrupted by various noises, including baseline wander (Below 1 Hz—low-frequency noise due to breathing, powerline interference (50HZ or 60 Hz from power lines), and muscle noise. Noises must be removed from signal before extracting features, as they can cause incorrect feature extraction, resulting in poor results.

Butterworth bandpass filters are commonly used. They consist of a passband and stopband with a maximally flattened frequency response Band without distorting the signal. The filter Normalizes The Cutoff Range through Nyquist frequency to avoid aliasing.


$$\omega_{low}=\;\frac{f_{low}}{f_{N}}\;,\;\omega_{high}=\;\frac{f_{high}}{f_{N}}\;$$

**Fig 3:**
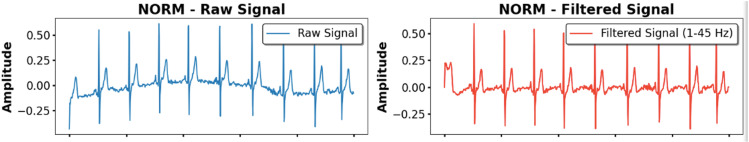
Signal filtering using butterworth bandpass signal to eliminate noise.

Here, fN stands for Nyquist frequency. A Butterworth filter of 1–45 Hz was applied to the dataset to reduce the noise frequency and adjust the baseline drift caused by it. (Fig 3) The choice of this frequency range ensures that the ECG waveform, including the P-QRS-T components, is retained. Since powerline noise lies above the selected cutoff, additional notch filtering was deemed unnecessary.

Muscle noise is coupled with the ECG signal and requires sophisticated filtering techniques. For this purpose, Discrete Wavelet Transform (DWT) is used [16]. The signal is decomposed into high-level and low-level components as it passes through various high-pass and low-pass filters. At each level, the signal is split into detail and approximation coefficients. The approximation coefficient (cA) is a low-frequency component that preserves the essential features and information of ECG beats. The detail coefficient (cD) is a high-frequency level component that preserves the shape information of the beats.

The signal is down-sampled to 8 levels to avoid redundancy in the dataset, allowing high-resolution analysis at various levels while iteratively decomposing the approximation coefficient to the desired level of decomposition. At the selected coefficient, the components are thresholded and used to reconstruct the signal without noise. The 8-level decomposition of DWT using the Daubechies wavelet is performed because of its resemblance with the QRS complex and high accuracy [17].

The function is carried out in three steps,

#### 1. Filter design.

Daubechies wavelet is considered the mother wavelet in DWT with the decomposition level 8, db8, and the length of the filter coefficient set at 16.

#### 2. Convolution.

High-pass and low-pass filters are applied to the signal. If the signal is represented as x[n], the low-pass filter as h[n], and the high-pass filter as g[n], then for j-level decomposition, the coefficients will be,


$$A_{j}\;\left[k\right]=\;\sum_{n}x\;\left[n\right]h\left[2k-n\right]\;$$

$$D_{j}\;\left[k\right]=\;\sum_{n}x\;\left[n\right]g\left[2k-n\right]\;$$

A_j_ [k] is the approximation coefficient, and D_j_ [k] is the detail coefficient for j-level decomposition.

#### 3. Downsampling.

**Fig 4:**
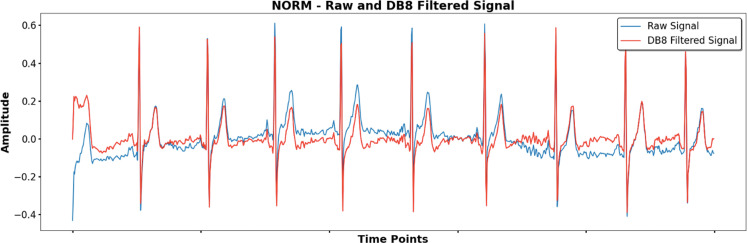
Applying db8 DWT to enhance signal-to-noise ratio.

The signal is down-sampled by a factor of 2, keeping every 2^nd^ sample resulting in a cleaner signal (Fig 4).

### D. QRS detection

**Fig 5:**
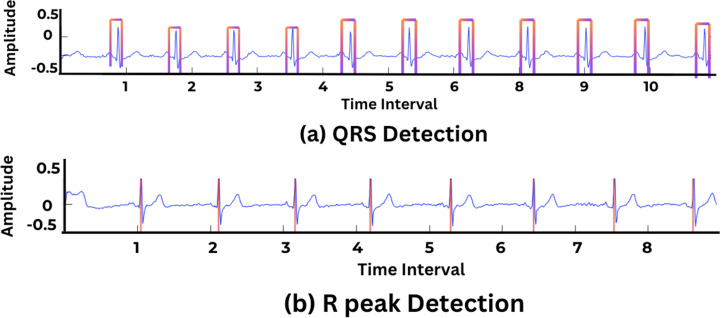
QRS detection in ECG signal using pan-tompkins algorithm with R-peak detection.

Each cardiac cycle consists of one QRS complex and has three characteristic points: Q, R, and S. It range from 0.06 to 0.10 seconds, during which the ventricular depolarization of heart chambers occurs. Accurately detecting the QRS complex within the cycle relies on efficient R-peak detection. The R-peaks are the baseline for detecting the QRS complex and extracting different morphological features. The Pan-Tompkins algorithm [18] in MATLAB, which is a sophisticated toolbox that allows R-peak detection using a four-step approach (Fig 5).

#### 1. Bandpass filtering.

Since the QRS segment is usually located within 5–15 Hz, a bandpass filter is applied to extract the QRS complex.


$$y\left[n\right]=\;\sum_{k=0}^{N}b_{k}x\left[n-k\right]-\;\sum_{k=1}^{M}a_{k}y\;[n-k]$$

For the input signal, x[n], b_k,_ and a_k_ are the filter coefficients for the filtered output signal y[n].

#### 2. Derivative filter.

The filter focuses on the high-frequency component that reflects the QRS complex.


y[n]=x[n]−x[n−1]

#### 3. Signal squaring.

By making all values positive, the signal enhances the QRS segment while reducing more minor variations.

#### 4. Signal average.

The signal is passed through a 0.150-second moving average filter to smooth the squared signal.


$$y\left[n\right]=\;\frac{1}{N}\sum_{k=0}^{N-1}x\;[n-k]$$

Here, N is the window length for the signal.

The following decision rules are incorporated to increase the result’s precision and accuracy.

1. **Fiducial Mark:** To localize the QRS complex at a timestamp with precision, weighted unit samples are generated at the maxima of the Moving Window Integration (MWI) output.
2. **Thresholding:** Two types of adaptive thresholding, Signal and Noise thresholding, are applied. The adaptive nature of the threshold allows it to capture varying conditions within the signal and distinguish between it and noise.
3. **Search back:** If there is a long pause in detecting the QRS complex, a search is initiated to identify any missed QRS complex to avoid the possibility of a false negative.
4. **Eliminating false detection:** Since the heart requires a refractory period before starting the second round of electrical activity, the chances of any false positive results are eliminated using the algorithm.
5. **Miscalculation:** To avoid miscalculation, the algorithm is adjusted to discriminate between the T-wave and QRS complex.
6. **Noise check:** To discard noise peaks within the signal, the algorithm applies the following check

T_currentpeak _< 360 ms and T_currentpeak _< 0.5 x mean_RR; classify as noise

### E. Feature extraction

The abnormal classes are reflected on the ECG through an altered waveform. Thus, morphological features are the predominant predictive features to be used for model training. The feature extraction process was designed with carefully defined search windows based on the R-peak, which serves as the anchor point for identifying other ECG waveform components. The Pan-Tompkins algorithm reliably detects the R-peak, allowing for precise localization of subsequent waves and intervals.

Given the variability across these classes, where different abnormalities can distort the waveform in specific ways, the search windows were adjusted dynamically to accommodate such variations. For each ECG record, after detecting the R-peak:

- **Time windows** were established before and after the R-peak to isolate the P wave (for PR and PQ intervals), Q wave, and S wave (for QRS duration). The width of these windows was tailored based on the typical durations of these intervals in normal and abnormal ECGs, ensuring that the feature extraction captured subtle variations in all five classes.
- **Amplitude extraction** for the P, Q, R, and T waves was achieved by locating the local maxima and minima within the respective time windows. For example, the window for detecting the P wave began well before the R-peak, whereas the windows for Q and S wave identification were centred around the R-peak. This method ensured the feature extraction was robust against class-specific waveform distortions, such as the widened QRS complex seen in conduction disturbances or the altered T wave in hypertrophy cases.

By defining these windows relative to the R-peak and adapting them to the specific class-based distortions, the feature extraction process could accurately capture the temporal (intervals) and spatial (amplitudes) characteristics of the ECG waveform. A total of 99 features extracted per ECG record are listed below;

#### • RR mean global.

The average time interval between consecutive R-peaks across all 12 leads reflects overall heart rate variability.

#### • ST elevation.

This measures the deviation of the ST segment from the baseline, calculated for each lead by measuring the upward deflection following the QRS complex.

#### • PR interval.

The time between the onset of the P wave and the beginning of the QRS complex measuring the atrioventricular conduction.

#### • PQ interval.

Similar to the PR interval, this interval measures the time from the start of the P wave to the onset of the Q wave, which reflects atrial conduction.

#### • QRS duration.

The duration of the QRS complex, indicating ventricular depolarization.

#### • QT interval.

The time from the start of the Q wave to the end of the T wave. This interval helps assess the overall duration of ventricular depolarization and repolarization.

#### • R, Q, and P amplitudes.

The peak amplitudes of the R, Q, and P waves measured across all leads.

#### • PR interval global.

The global PR interval, calculated by averaging the PR intervals across all 12 leads.

#### • PQ interval global.

The global PQ interval, derived by averaging the PQ intervals across all leads.

**Fig 6:**
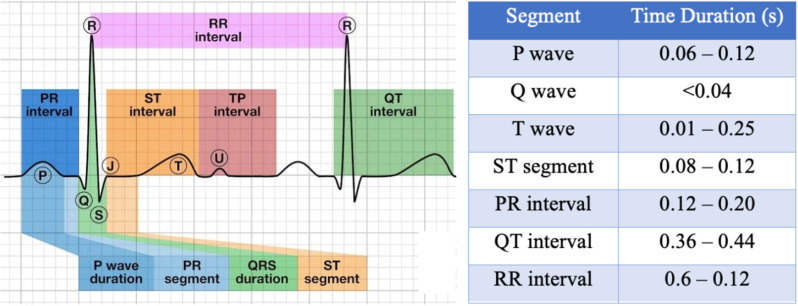
Location and duration of each segment in the ECG waveform.

Fig 6 represents the location and time duration of P-QRS-T amplitudes and intervals of a Normal ECG waveform.

### F. Class balance

**Fig 7:**
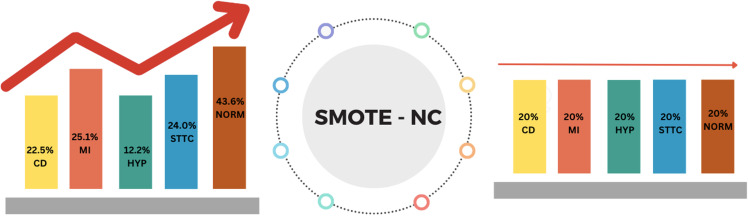
Balancing the dataset with SMOTE-NC to prevent model bias.

The PTB-XL dataset contains a significant class imbalance, with the NORM class having a large number of ECG records, while Hypertrophy (HYP) is severely underrepresented, with only 530 instances. This imbalance could result in model bias, where the majority class dominates the classification process, leading to poorer performance on minority classes. To address this challenge, SMOTE-NC (Synthetic Minority Over-sampling Technique for Nominal and Continuous variables) is applied to balance the dataset. (Fig 7)

SMOTE-NC creates synthetic instances for the minority classes by interpolating between existing instances. Unlike traditional oversampling or undersampling methods, SMOTE-NC generates new instances that preserve the original feature distribution, making it especially effective for datasets with both continuous (ECG signal features) and categorical variables (class labels), such as the PTB-XL dataset. By upsampling the minority classes, SMOTE-NC ensures that all five classes, Normal (NORM), Myocardial Infarction (MI), Hypertrophy (HYP), Conduction Disturbance (CD), and ST-T Changes (STTC), are equally represented during training.

This step is crucial for improving the model’s ability to classify minority classes without bias. SMOTE-NC is applied to the training, validation, and test sets to ensure that class balancing occurr across all phases of the model evaluation. By expanding the representation of the minority classes, the model is prevented from being biased toward the NORM class, which would otherwise dominate the learning process.

### G. Classification

**Fig 8:**
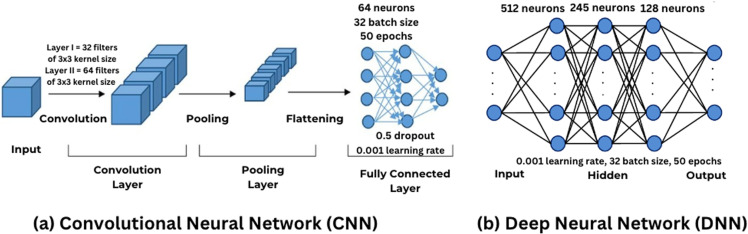
Architecture of CNN vs. DNN models.

DL models have gained popularity among researchers after the introduction of the backpropagation algorithm [19]. This research uses two models: CNN and DNN. CNN performs well with time-series signals because of the windowed convolutional that creates a receptive field. This acts as a sensory area that enables a neuron [20]. On the other hand, DNN has shown excellent results with an end-to-end deep learning approach [21]. Both CNN and DNN utilize neurons in their hierarchical structure. (Fig 8)

#### Convolutional Neural Network (CNN).

A CNN is a feedforward neural network with convolutional, ReLU, pooling, flattening, and dense layers, specialized for a grid-like topology [22]. ECG can be effectively trained with CNN, serving as 1-D time series data. The CNN architecture used consists of:

- **Convolution:** Two sets of convolutional layers, the first with 32 filters of 3x3 kernel size and the second with 64 filters of 3x3 kernel size, followed by ReLU activation.
- **Pooling:** Pooling layers reduce the dimension of the feature map, preventing overfitting.
- **Flattening:** The output of pooling layers is flattened and set as input for dense layers.
- **Dense Layer:** Consists of 64 neurons using ReLU activation and a dropout of 0.5 for efficiency.
- **Output Layer:** Uses the Softmax algorithm to classify signals into various class probabilities.

The CNN was trained using the Adam optimiser with an initial learning rate of 0.001, which was decayed based on validation loss. The batch size was set to 32, and the model was trained for 50 epochs. A 5-fold cross-validation scheme ensured the model generalized well to unseen data. Additionally, early stopping was implemented to prevent overfitting during training.

#### Deep Neural Network (DNN).

A DNN is a densely connected structure with input, hidden, and output layers [23]. The architecture includes:

- **Hidden Layers:** Consisting of 512, 245, and 128 neurons.
- **Optimization Techniques:** It includes a StandardScaler for performance optimization, ReLU activation for non-linearity, Batch Normalization for stability, Dropout to prevent overfitting, and Softmax activation in the output layer for class probability activation.

The DNN also utilized the Adam optimizer with an initial learning rate of 0.001, adjusted during training using a learning rate scheduler. A batch size of 32 was used with 50 epochs of training. Like the CNN, 5-fold cross-validation was used, and early stopping was applied to avoid overfitting.

After training, the models were tested, and the performance was evaluated by comparing the predicted and true labels. A confusion matrix was created along with other performance metrics. It includes,

1. **Sensitivity:** It is also called Recall or True Positive Rate. It measures the model’s ability to correctly identify the True positive labels within the dataset. High recall means the model correctly identified the right labels with low error chances.

$$Recall=\;\frac{True\;Positives\;(TP)}{True\;Positives\;\left(TP\right)+False\;Negatives\;(FN)}$$

1. **Specificity:** It is also called a True Negative Rate. It measures the correct negative instances correctly identified by the model. High specificity means the model correctly identified negative instances with low false positives.

$$Specificity=\;\frac{True\;Negatives\;(TN)}{True\;Negatives\;\left(TN\right)+False\;Positives\;(FP)}$$

1. **Accuracy:** It is a comprehensive measurement of the model’s ability to identify both True Positive and True Negative instances correctly. High accuracy means the model can effectively identify both instances with minimized error.

$$Accuracy=\;\frac{True\;Positives\;\left(TP\right)+True\;Negatives\;(TN)}{Total\;Number\;of\;Instances}$$

$$Accuracy=\;\frac{TP+TN}{TP+TN+FP+FN}$$

1. **Precision:** It is also called Positive Predictive Value. It measures the prediction of correctly identifying the positive labels as positive. High precision means that when a model predicts positive labels, it will be a true positive.

$$Precision=\;\frac{True\;Positives\;(TP)}{True\;Positives\;\left(TP\right)+False\;Positives\;(FP)}$$

1. **F1 score:** It is the harmonic mean of both sensitivity and precision. It provides a single subset to balance both measures.

$$F1\;Score=\;2\;x\;\frac{Precision\;x\;Recall}{Precision+Recall}$$

## Results

**Fig 9:**
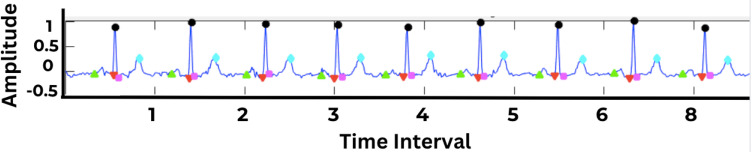
Detection of PQRST amplitudes in ECG waveform.

After filtering the dataset to include records with only one unique label, the final dataset consisted of 17,232 raw ECG records. These records were pre-processed using a Butterworth bandpass filter and Db8 DWT to remove noise and correct baseline wander. Following pre-processing, the morphological features were extracted (Fig 9), labeled, and upsampled to address the class imbalance. The dataset was split into the training and testing sets with a ratio of 80–20% and fed into the CNN and DNN models with a 5-fold loop and 50 iterations. This allowed the model to pick every signal to analyze and learn patterns robustly. The performance metrics allowed for the comparison ofthe model’s output.

**Fig 10:**
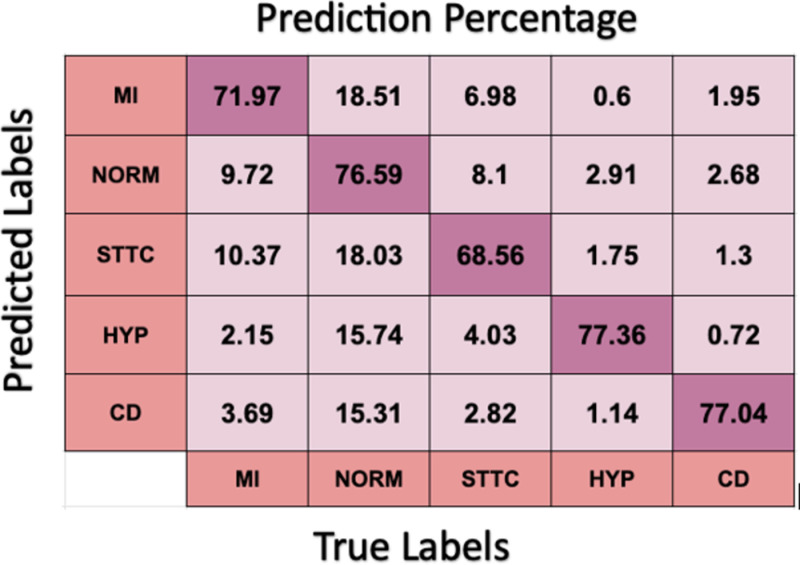
CNN performance in predicting labels across five classes.

CNN achieved a mean accuracy of 81% ± 0.03 on training data. The confusion matrix (Fig 10) indicated that the model exhibited strong performance in classifying Hypertrophy (HYP) and Conduction Disturbance (CD). The distinct morphological features associated with these classes—such as prolonged QRS duration in CD and exaggerated wave amplitudes in HYP—likely contributed to higher sensitivity for these conditions. The CNN achieved 74.30% sensitivity and 93.59% specificity on the test data, with the confusion matrix showing some misclassification between similar classes, such as between ST-T Changes (STTC) and Myocardial Infarction (MI). This misclassification can be attributed to the subtle similarities in the ST-segment deviations and QRS patterns in these conditions.

The F1 score, as shown in Table 1 for HYP, was significantly higher than for other classes, demonstrating CNN’s ability to distinguish hypertrophy.

**Table 1:** Performance metrics of CNN.

	Precision	Recall	F1 Score	Support
**MI**	0.74	0.72	0.73	1848
**NORM**	0.53	0.77	0.62	1790
**STTC**	0.75	0.69	0.72	1775
**HYP**	0.92	0.77	0.84	1811
**CD**	0.92	0.77	0.84	1842

**Fig 11:**
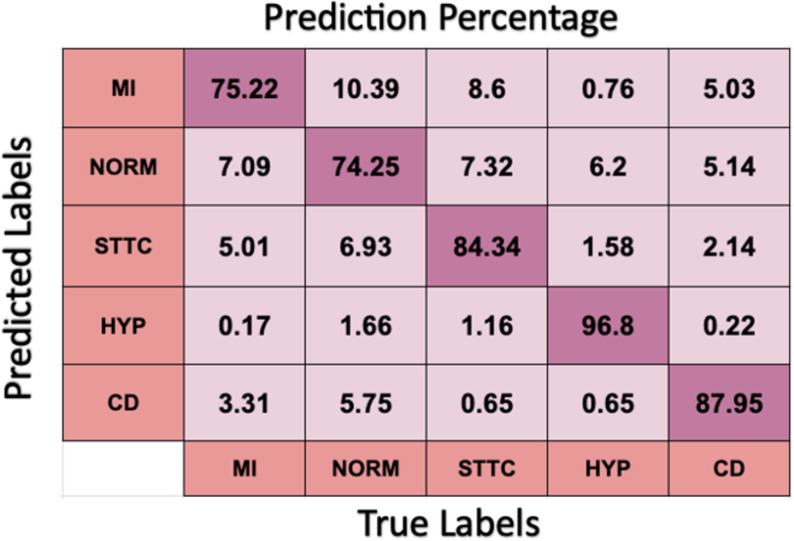
DNN performance in predicting labels across five classes.

The DNN model exhibited better performance, achieving a mean accuracy of 84% ± 0.01 on the training data. During testing, the DNN model maintained robust performance with an accuracy close to training data, yielding 83.71% sensitivity and 95.93% specificity. The class-wise analysis indicated that the DNN, similar to CNN, performed exceptionally well in detecting Hypertrophy (HYP) and Conduction Disturbance (CD) (Fig 11). However, it also showed improved performance in distinguishing between ST-T Changes (STTC) and Myocardial Infarction (MI), a key area where CNN showed higher misclassification rates.

The F1 score for STTC was significantly improved with DNN, as shown in Table 2, suggesting better generalization across the five classes, particularly for classes with similar waveform abnormalities.

**Table 2:** Performance metrics of DNN.

	Precision	Recall	F1 Score	Support
**MI**	0.83	0.75	0.79	1848
**NORM**	0.75	0.74	0.74	1790
**STTC**	0.81	0.84	0.83	1775
**HYP**	0.91	0.97	0.94	1811
**CD**	0.88	0.88	0.88	1842

The DNN’s performance in distinguishing Normal (NORM) from abnormal classes remained consistent. The confusion matrix showed a high true negative rate for NORM cases, reflecting the DNN’s ability to classify normal ECG signals correctly. This performance reinforces the DNN’s robustness in handling the imbalanced dataset, aided by the SMOTE-NC technique to balance minority classes like HYP.

To further highlight the contribution of this work, Table 3 presents a comparison of performance metrics from the current study and previous works that used the same PTB-XL dataset. This comparison emphasizes the pivotal role of the current study’s performance, particularly for minority classes after applying SMOTE-NC for class balancing.

**Table 3:** Comparison of performance metrics across studies.

Study	Class Handling Technique	Model	Accuracy	Average F1
Smigiel et al. 2021 (a)	Class imbalance	CNN	0.72	0.611
Smigiel et al. 2021 (a)	Class imbalance	CNN with Entropy features	0.76	0.68
Smigiel et al. 2021 (b)	Class imbalance	CNN with Raw signal and its entropy features	0.75	0.64
Smigiel et al. 2021 (b)	Class imbalance	CNN with QRS and its entropy features	0.76	0.68
Palczynski et al. 2022	Class imbalance	FSL-proximity based with extracted QRS	0.71	0.63
Palczynski et al. 2022	Class imbalance	Softmax-based classification with extracted QRS	0.75	0.67
Current study	Class balanced by SMOTE-NC	CNN with PQRST features	**0.81**	**0.75**
Current study	Class balanced by SMOTE-NC	DNN with PQRST features	**0.84**	**0.83**

## Discussion

This study applied an advanced approach to heart disease classification using ECG signals, combining Butterworth bandpass filtering with Discrete Wavelet Transform (DWT) for noise reduction and signal enhancement. The research achieved cleaner signals by employing both methods, improving the accuracy of extracted morphological features, such as P-QRS-T intervals and amplitudes. Once fed into CNN and DNN models, these features significantly improved classification accuracy, with DNN showing consistently higher performance.

This research represents a notable advancement in multi-class ECG classification compared to previous studies. Using simpler models like LDA and Navies Bayes, Bhoi et al. [24] achieved 100% accuracy in detecting ischemia and arrhythmia. However, their study was limited to a small dataset and fewer episodes of disease detection. Similarly, Marinho et al. [25] applied techniques like Fourier Transform (FT) and High-Order Statistics (HOS). In contrast, He et al. (26) applied Deep Residual Networks (DRN) and BiLSTM to the MIT-BIH arrhythmia dataset, achieving reasonable accuracy. However, their method showed poor performance when applied to more complex ECG signals and diverse conditions. Anbalagan et al. [26,27]utilized different ML and DL models capturing time-frequency and wavelet-based features yielding great results; however, the work focused solely on Atrial fibrillation.

Compared with studies such as those by Sharma et al. [28] and Yao et al. [29], who focused on Fourier-Bessel and ATI-CNN techniques, this research offers a more holistic approach. While Sharma and Yao achieved accuracies close to 90%, their models were limited to binary classifications and a small subset of diseases. The multi-class classification framework adopted in this study, with five distinct heart conditions, Normal (NORM), Myocardial Infarction (MI), Hypertrophy (HYP), Conduction Disturbance (CD), and ST-T Changes (STTC), ensured a more comprehensive understanding of heart diseases. The inclusion of the SMOTE-NC technique for addressing class imbalance further strengthened the model’s performance, particularly in underrepresented classes like HYP and CD, which is a significant improvement over earlier works that did not effectively resolve class imbalances, such as those by Hasan and Bhattacharjee [30]. Similarly, Palczynski et al. [10] and Smigel et al., [3] demonstrated the challenges of achieving high performance in these minority classes due to class imbalance. For instance, Palczynski et al. reported accuracies of 2.5% for HYP and 11.4% for MI, while Smigel et al. achieved 1.3% for HYP and 10.2% for MI. In contrast, this study, by incorporating SMOTE-NC, demonstrated a significant improvement, with both CNN and DNN models achieving over 75% accuracy for MI and HYP, thereby effectively addressing the class imbalance problem and improving the overall model performance. SMOTE-NC was specifically chosen for its ability to create synthetic samples by interpolating between existing instances of the minority classes. This technique effectively increases the diversity of the training data, which helps the model learn from a more representative dataset. Unlike traditional methods, which often replicate minority class instances, SMOTE-NC introduces new, plausible data points that prevent overfitting and enhance the model’s ability to generalize to unseen data. The improvement in model performance, especially for minority classes such as HYP and CD, clearly highlights the effectiveness of SMOTE-NC in enhancing classification accuracy. By ensuring a more balanced representation across all five classes, SMOTE-NC allowed the deep learning models to better handle the complexities of multi-class ECG classification, ultimately resulting in a more robust and reliable model.

A key strength of this research lies in its use of DNN, which outperformed CNN with an accuracy of 84% ± 0.01. The superior performance of DNNs can be attributed to their ability to learn and represent complex patterns in the ECG signals more effectively than CNNs [31]. One challenge common in deep learning, including this study, is the reduced interpretability of DNN models. While CNN offers more localized feature extraction, the complexity of DNN introduces the “black-box” issue, complicating clinical application. Similar challenges were observed in other works, such as Bonab et al.. [32]. Future work should explore the integration of Explainable AI (XAI) techniques to improve model transparency, enabling better interpretability in clinical applications.

Additionally, the computational complexity of training and deploying DNN models may present challenges for real-time deployment in clinical settings, where resource efficiency is crucial. This is a trade-off observed in previous works, such as those by Mohonta et al. [33] and Oktivasari et al. [34], where simpler models were used to achieve faster performance at the cost of classification depth and accuracy. Despite these challenges, the models used in this study demonstrate a clear improvement over earlier methods, especially in handling the complexities of multi-class ECG classification.

This study significantly contributes to ECG-based disease detection, improving previous research’s limitations by utilizing a larger, more diverse dataset, advanced feature extraction techniques, and powerful deep learning models. These advancements lay a strong foundation for future work to enhance model interpretability and reduce computational complexity, paving the way for more effective real-time clinical use.

## Conclusion

This research presents a significant advancement in the classification of cardiovascular diseases (CVDs) using ECG signals through the application of deep learning techniques, namely Convolutional Neural Networks (CNNs) and Deep Neural Networks (DNNs). By combining Butterworth bandpass filtering with Discrete Wavelet Transform (DWT) for noise reduction, the study successfully extracted key morphological features such as P-QRS-T intervals and amplitudes, which were critical in improving classification accuracy. The results demonstrate the DNN model’s superiority, achieving an accuracy of 84% ± 0.01, particularly in distinguishing between complex heart conditions like Hypertrophy (HYP) and Conduction Disturbance (CD).

Unlike previous studies, which were either limited by small datasets or binary classifications, this research adopted a multi-class framework. It successfully addressed class imbalances using SMOTE-NC and provided a more comprehensive understanding of ECG-based CVD detection. While DNN outperformed CNN in this study, challenges related to model interpretability and computational complexity remain, particularly for real-time clinical applications.

Future research will focus on integrating Explainable AI (XAI) techniques to improve model transparency and enable better interpretability in clinical settings. This will be crucial for overcoming the “black-box” nature of DNNs and making the model more accessible for healthcare professionals.

Additionally, to address the computational demands of DNN models, future research will explore strategies to reduce model complexity while maintaining high performance. This may include the implementation of lightweight models or hybrid techniques that balance computational efficiency with classification accuracy. At the same time, we plan to test more advanced models such as ResNet, Inception V3, and GRU, optimizing their hyperparameters to assess their potential for improving classification accuracy. By comparing their performance with the current CNN and DNN models, we aim to determine how these advanced models can enhance accuracy without sacrificing computational efficiency.

Overall, this study makes a substantial contribution to ECG-based disease detection by improving the classification accuracy of complex cardiovascular conditions and addressing key challenges in the field. The study lays a strong foundation for real-time, multi-class ECG classification, and the ongoing exploration of interpretability, computational efficiency, and generalization will only strengthen the impact of this work, supporting early intervention and improving clinical decision-making.
